# Exploring the impact of grazing on fecal and soil microbiome dynamics in small ruminants in organic crop-livestock integration systems

**DOI:** 10.1371/journal.pone.0316616

**Published:** 2025-01-17

**Authors:** Sejin Cheong, Kimberly Aguirre-Siliezar, Sequoia R. Williams, Amélie C. M. Gaudin, Paulo Pagliari, Michele T. Jay-Russell, Roselle Busch, Elizabeth A. Maga, Alda F. A. Pires

**Affiliations:** 1 Department of Population Health and Reproduction, School of Veterinary Medicine, University of California-Davis, Davis, Davis, California, United States of America; 2 Department of Plant Sciences, University of California-Davis, Davis, Davis, California, United States of America; 3 Department of Soil, Water and Climate, College of Food, Agriculture and Natural Resources Sciences, University of Minnesota, Minneapolis, Minnesota, United States of America; 4 Western Center for Food Safety, University of California-Davis, Davis, Davis, California, United States of America; 5 Department of Animal Science, University of California-Davis, Davis, Davis, California, United States of America; Cornell University, UNITED STATES OF AMERICA

## Abstract

In integrated crop-livestock systems, livestock graze on cover crops and deposit raw manure onto fields to improve soil health and fertility. However, enteric pathogens shed by grazing animals may be associated with foodborne pathogen contamination of produce influenced by fecal-soil microbial interactions. We analyzed 300 fecal samples (148 from sheep and 152 from goats) and 415 soil samples (272 from California and 143 from Minnesota) to investigate the effects of grazing and the presence of non-O157 Shiga toxin-producing *Escherichia coli* (STEC) or generic *E*. *coli* (gEc) in fecal and soil microbiomes. We collected samples from field trials of three treatments (fallow, a cover crop without grazing (non-graze CC), and a cover crop with grazing (graze CC)) grazed by sheep or goats between 2020 and 2022. No significant differences in non-O157 STEC prevalence were found between pre- and post-grazing fecal samples in either sheep or goats. However, gEc was more prevalent in graze CC soils compared to fallow or non-graze CC soils. Alpha diversity was influenced by the species of grazing animals and the region, as sheep fecal samples and soil from the California trials had greater alpha diversity than goat fecal samples and soil from the Minnesota trials. Beta diversity in sheep fecal samples differed by the presence or absence of non-O157 STEC, while in goat fecal samples, it differed between pre- and post-grazing events. Actinobacteria was negatively associated with non-O157 STEC presence in sheep fecal samples and decreased in post-grazing goat fecal samples. Grazing did not significantly affect soil microbial diversity or composition, and no interaction was observed between post-grazing fecal samples and the graze CC soil. The results suggest that soil contamination by foodborne pathogens and microbiome dynamics in ICLS are influenced by grazing animal species and regional factors, with interactions between fecal and soil microbial communities having minimal impact.

## Introduction

Integrated crop-livestock systems (ICLS) utilize livestock to graze on cover crops or residue crops before a field is replanted with produce intended for human consumption [1]. In ICLS, grazing livestock are used to fertilize the soil and manage cover crops, reducing the need for supplemental fertilizer or labor for mowing, thereby lowering production costs [2]. Livestock integration in ICLS provides other benefits, such as increased crop yields and improved environmental sustainability by promoting soil microbial biomass and biodiversity, nutrient recycling, and insect pest management [1–3]. However, one of the concerns with the ICLS practice is the potential contamination of produce crops with foodborne pathogens shed by livestock.

Livestock are known to be natural reservoirs for foodborne pathogens, posing a major concern in the agricultural industry as they can be entry points for pathogens into the food supply [4,5]. Most livestock carriers are asymptomatic, frequently shedding pathogens in their feces without showing signs of illness [6]. Among foodborne pathogens, Shiga toxin-producing *E*. *coli* (STEC) is currently a public health concern due to its potential to cause serious health problems, such as hemorrhagic colitis and hemolytic uremic syndrome [7]. Recently, outbreaks caused by STEC have been reported in various types of fresh produce as the consumption of vegetables increases [8,9]. Nonetheless, there is limited reseach assessing the foodborne pathogen shed from small ruminants, such as sheep and goats [10,11].

Current literature on fecal microbiomes suggests that animal species and external factors such as diet, husbandry, and environmental management influence pathogen shedding and fecal microbial composition [12–14]. An experimental study in sheep reported that a diet change from alfalfa to pasture grass affected the fecal shedding of *E*. *coli* O157:H7 [15]. Moreover, dietary differences due to husbandry management and starch-based feeding practices affected the abundance of fecal microbial taxa in cattle [13,16]. Additionally, the taxa and metagenome composition of cattle fecal microbial communities were associated with the onset of shedding foodborne pathogens, such as *Salmonella* and *E*. *coli* O157:H7 [17,18].

The contamination of soil with foodborne pathogens through fecal deposition is relevant to the diversity of the soil microbiome on farms [19–21]. When raw manure is applied to soil, it is known to increase soil microbial biomass and abundance by activating underrepresented soil-borne taxa [22,23]. Indeed, organic produce farms, which often enrich soil with biological soil amendments of animal origin (e.g., compost or manure), have greater bacterial diversity and higher levels of organic matter than farms with conventionally managed soils [23,24]. An experimental study in the Netherlands showed that the survival of *E*. *coli* O157:H7 was negatively associated with the diversity of the soil community [21]. Less diverse soil communities have been found to exhibit larger changes in their resident bacterial communities when exposed to a foreign microbe [20,25]. For instance, when foodborne pathogens such as *L*. *monocytogenes* and *Salmonella* were inoculated into soils from different production systems, soil with higher organic matter managed with cover crops and animal-based compost showed a steeper decline in pathogen survival than conventional soil without these practices [19]. Therefore, the probability of raw manure application causing pathogen contamination in soil may be linked to lower indigenous soil microbiome diversity. However, this aspect has not been investigated in ICLS involving short-term grazing with small ruminants.

This study was motivated by the lack of information regarding the role of small ruminants in enteric pathogen shedding while grazing and the intricate interplay between fecal and soil microbiomes under ICLS practices. Specifically, we aimed to investigate the prevalence of non-O157 STEC or generic *E*. *coli* (gEc) in fecal and grazed soil samples collected from ICLS field trials (2020–2022) conducted in California (CA) and Minnesota (MN). Additionally, we evaluated the associations between fecal microbiomes and the presence of non-O157 STEC in sheep and goats pre- and post-grazing. We expected that grazing in ICLS would increase the diversity of the soil microbiome and influence the soil’s pathogen contamination.

## Materials and methods

### Study design and sample collection

Three replicated field trials were conducted in two states (CA and MN). The CA trials were conducted over three years (2020–2022) at the Russell Ranch Sustainable Agriculture Facility, University of California, Davis (UC Davis) (38° 32’ 36.87", -121° 52’ 11.89"). The MN trial was carried out over two years (2021–2022) at the University of Minnesota Southwest Research and Outreach Center (44° 14’ 29.4858", -95° 19’ 1.5312"). A randomized complete block design with four replicates was conducted in both states, with a random allocation of three treatments in each block—fallow ground as a control, a cover crop tilled without grazing (non-graze CC), and a cover crop grazed by sheep or goats (graze CC). In CA, we conducted two distinct field trials: one with tomatoes as the produce crop (2020–2021) (CA Trial 1) and one with a spinach/cucumber crop rotation (2021–2022) (CA Trial 2). In MN, the trial was conducted from 2021 to 2022 with a spinach/cucumber crop rotation (MN Trial 3), as in CA Trial 2. The cover crop mix was adapted to each state: it consisted of cereal rye, crimson clover, and daikon radishes in CA, while the mix in MN comprised winter rye, berseem clover, and daikon radishes. For grazing, the CA trials used a sheep flock composed of Suffolk, Hampshire, Dorset, and crossbreeds aged from 1–5 years old, while the MN trial used a goat herd composed of Spanish meat goat breeds aged from yearlings to 10 years old. The number of grazing animals and duration of grazing were determined based on the quantity of cover crop biomass each year. In the CA trials, a flock of 25–80 sheep grazed once or twice annually, with each grazing event lasting for 1–3 days between February and April. In the MN trial, a herd of 40–170 goats grazed twice annually for 3 days in October/November and April/May. This study was approved by the Institutional Animal Care and Use Committee of the University of California, Davis (IACUC #22700) and the University of Minnesota (IACUC #2008-38348A).

Fecal samples were collected pre- and post-grazing (20–28 samples each). Pre-grazing samples were collected from the barn floor in CA or the trailer floor in MN before the animals were moved to the grazing plots. Post-grazing samples were collected from the fields immediately after grazing events. In the CA trials, control fecal samples (8 samples per grazing event) were collected from sheep in the same herd that were not moved to the grazing plots and received the same pre-trial diet as the grazing sheep. Soil samples (36–72 samples per sampling day) were collected monthly after the last grazing event for up to 120- and 150-days post-grazing (DPG). Additional soil samples were collected from the spinach/cucumber fields in CA and MN 7 DPG. Detailed protocols for the grazing scheme and sample collection of soil and feces are described in Cheong et al. (2024) [26]. Samples were processed within 48 h after collection for microbial analysis to assess the presence of STEC or gEc. Approximately 10g of each collected sample was stored at -20°C before DNA extraction. DNA was extracted from all of the collected fecal samples and a random selection of one-third of the soil samples from each block (i.e., 12–24 soil samples per sampling day) to perform 16S rRNA gene sequencing. The total number of samples used for DNA extraction included 347 fecal samples (187 sheep fecal samples and 160 goat fecal samples) and 457 soil samples (306 soil samples from CA and 151 from MN).

### Microbial analyses

Pre- and post-grazing fecal samples were examined for the presence of non-O157 STEC, and soil samples were assessed for the presence of non-O157 STEC and gEc as indicators of fecal contamination.

Each fecal or soil sample (10 g per fecal sample and 30 g per soil sample) was enriched in a 24 oz. Whirl-Pak bag filled with 90ml or 270ml of tryptic soy broth (TSB) (BD BactoTM, Heidelberg, Germany), respectively, to detect non-O157 STEC or gEc. For the TSB enrichment, samples were incubated at 25°C for 2 h followed by 42°C for 8 h with 50 rpm shaking, then held at 6°C without shaking in a Multitron programmable shaking incubator (Eppendorf, Hauppauge, NY, United States).

To isolate non-O157 STEC, 1 mL of enriched TSB sample was put into a tube with 9mL of modified enterohemorrhagic *E*. *coli* (mEHEC) selective media (Biocontrol, Bellevue, WA, United States) and incubated for 12 h at 42°C [27]. Then, mEHEC solution was streaked onto ChromSTEC agar (CHROMagarTM, Paris, France) using a 10 μL inoculation loop and incubated for 24 h at 37°C. Presumptive positive isolates (i.e., purple colonies that fluoresced under ultraviolet light) on ChromSTEC agar were re-streaked onto secondary and tertiary ChromSTEC agar. A final pure presumptive positive colony was streaked onto tryptic soy agar (TSA) for confirmation as non-O157 STEC with a standard polymerase chain reaction (PCR) assay targeting the *stx1* and *stx2* genes [28].

Ten μL from each TSB bag was streaked onto CHROMagar *E*. *coli* (ECC) (CHROMagar Microbiology, Paris, France), followed by incubation for 24 h at 37°C to determine the presence of gEc. After re-streaking presumptive positive colonies (i.e., blue colonies) onto secondary and tertiary ECC plates, the pure isolates on TSA were confirmed as gEc by PCR targeting the *uspA* gene [29].

### DNA extraction and 16s rRNA gene sequencing

DNA was extracted from fecal (≤ 0.15g) and soil (≤ 0.25g) samples using the Quick-DNA Fecal/Soil Microbe Miniprep Kit #D6010 (Zymo Research, Irvine, CA), following the manufacturer’s instructions. The extracted DNA’s concentration and purity (A260/280 as 1.8–2.0) were quantified using a NanoDrop (Thermo Fisher Scientific, Wilmington, DE). Fecal samples with DNA concentrations of less than 10 ng/uL and soil samples with DNA concentrations of less than 4.5 ng/uL after DNA extraction were excluded from further steps. The V4 region of bacterial 16S rRNA genes was amplified using a unique 8 bp barcode attached to each forward primer per sample. The forward and reverse primers used were F515 (5′- GTGTGCCAGCMGCCGCGGTAA- 3′) and R806 (5′ - GGACTACHVGGGTWTCTAAT- 3′) [30]. Each sample was amplified by PCR in triplicate (25ul each) using GoTaq Hot Start Colorless Master Mix 2X (Promega, Madison, WI). PCR was run with the following conditions to amplify the DNA: initial denaturation at 94°C for 3 mins, 30 or 35 cycles of denaturation at 94°C for 45 secs (30 cycles for feces and 35 cycles for soil), annealing at 55°C or 50°C for 1 min (55°C for feces and 50°C for soil), elongation at 72°C for 90 secs, and final extension at 72°C for 10 min. The barcoded amplified triplicates were combined and visualized via electrophoresis using a 2% agarose gel. All samples were pooled based on the density of gel bands, which was determined using VisionWorks software. The pooled samples were purified using QIAquick^®^ Gel Extraction Kit (Qiagen, Valencia, CA) and assessed by a bioanalyzer for quality. Sequencing of the pooled samples was performed on the Illumina MiSeq PE250 platform at the University of California Davis Genome Center DNA Technologies Core. The total number of samples submitted for sequencing was 306 fecal and 423 soil samples, run as three different batches.

### Library processing, taxonomy assignment, and filtering

Raw sequence data processing and taxonomy assignment were performed using QIIME2 (v.2023.9) [31]. Raw paired-end reads were demultiplexed, and then linkers and barcodes were trimmed. The first 20 bases of the reads were trimmed off and truncated at 200 bp for each forward and reverse sequence and then merged for denoising with the DADA2 pipeline [32]. An amplicon sequence variant (ASV) table was created after removing chimeras. Taxonomy was assigned with naïve Bayes classifiers trained on the SILVA reference database v.138 [33]. Phylogenetic trees were generated using FastTree and MAFFT alignment after merging taxonomy data and ASV tables for each type of sample (fecal and soil) as the raw sequence data of all samples were sequenced in three lanes. The ASV tables, phylogenetic trees, taxonomy data generated by QIIME2, and metadata were imported into R software (v.4.2) using the phyloseq package (v.1.42) [34]. Samples with sequencing depth under 10 reads, taxonomy classified as non-bacterial (i.e., Archaea or Eukarya), and mitochondria or chloroplasts were removed before assessing the microbial communities. After filtering, the final feature tables used for further statistical analyses had 3,022 ASVs for the 300 fecal samples (148 sheep and 152 goat samples) and 5,361 ASVs for the 415 soil samples (272 soil samples from CA and 143 from MN).

### Statistical analysis

Descriptive statistics were used to summarize the prevalence (by proportion) of fecal and soil samples positive for non-O157 STEC or gEc by sample type, treatment, type of grazing animal, and year. A two-proportion t-test was used to compare the proportions between groups (e.g., post-grazing fecal samples from sheep and goats).

We investigated the following factors in fecal samples to further evaluate their effects on microbial composition: the type of grazing animal (sheep or goats), year, pre- and post-grazing status, and presence of non-O157 STEC. Additionally, fecal samples from the CA trials’ grazing and control groups were compared. For the soil samples, we investigated the effects of treatment (fallow, non-graze CC, and graze CC), the sampling day (DPG), state, and the presence of non-O157 STEC and gEc. The soil dataset was split into two groups for the analysis according to the type of produce crop(s): tomatoes (2020–2021)—CA Trial 1, and spinach/cucumbers (2021–2022)—CA Trial 2 and MN Trial 3.

Alpha and beta diversity were used to assess the microbiome composition in fecal and soil samples separately. Four types of alpha-diversity metrics were calculated to describe within-sample diversity: the Chao1, Shannon, Simpson, and Faith’s phylogenetic diversity (PD) indices. The alpha-diversity metrics were compared using a t-test between two groups or ANOVA for more than three groups. The Bonferroni pairwise t-test was used as a post-hoc test for significant ANOVA results [35]. Two types of beta-diversity metrics were used to assess the differences in microbial community composition between samples: Bray-Curtis dissimilarity takes into account abundances (count data), whereas unweighted UniFrac considers the phylogenetic distances (presence/absence) of the microbes in samples. Visualization was performed with Bray-Curtis dissimilarity using principal coordinate analysis (PCoA) after aggregating the rare taxa at the genus level with a detection threshold of 0.1% and a prevalence greater than 5%. In addition to Bray-Curtis dissimilarity, we used unweighted UniFrac at the ASV level without filtering for prevalence to observe the effects of explanatory variables on changes in microbial communities using permutational multivariate ANOVA (PERMANOVA) in the *vegan* package of R software (v.4.2) [36]. A *p*-value of < 0.05 was considered significant, and pairwise comparisons were performed for significant results in PERMANOVA.

We analyzed microbiome compositions with Bias Correction 2 (ANCOM-BC2) to determine differentially abundant taxa between groups based on the considered effects, using the *ANCOM* package (v. 2.5.0) in R software. The analysis applied linear regression models after (natural) log transformation of the observed counts of taxa, adding a series of pseudo-counts (0.01–0.5) to zero counts of each taxon for sensitivity analysis to prevent inflated false positive rates [37,38]. All differential abundance analyses were performed at the phylum and family levels after agglomerating the phyloseq data at the genus level. Taxa with a prevalence of less than 10% were excluded by default. The Benjamini-Hochberg adjustment was used to calculate adjusted *p*-values, and an adjusted *p*-value < 0.05 was considered significant.

Lastly, we evaluated the core microbiomes and the correlation between post-grazing fecal samples and soil samples collected from the graze CC treatment plots. Core microbiota analysis was conducted with a detection threshold of 0.1% and a prevalence greater than 50% within each sample at the ASV level using the “core” function in the *microbiome* package (v.1.20.0) [39]. Sparse estimation of linear correlations among microbiomes (SECOM) was performed with the *ANCOM* package’s (v. 2.5.0) “secom_linear” function in R software. Pearson correlation was examined at the phylum level, with a threshold of pairwise correlation set at 0.3. Based on the matrix result of the taxon-to-taxon co-occurrence pattern, pairs of taxa where the number of nonzero samples was less than 10 were excluded from the correlation calculation.

## Results

### Fecal samples

#### Prevalence of non-O157 STEC in fecal samples

Table 1 summarizes the prevalence of non-O157 STEC in fecal samples collected from sheep and goats from the CA and MN trials. There was no statistically significant difference (*p* = 0.8) between the non-O157 STEC prevalence in the post-grazing fecal samples from sheep (26.5%, 18/68) and goats (23.4%, 18/77). Additionally, there was no significant difference in the non-O157 STEC prevalence between the control (35.0%, 14/40) and the grazing group (i.e., the sum of the pre- and post-grazing groups; 26.9%, 29/108) in sheep, nor between pre- and post-grazing groups in either sheep and goats.

**Table 1:** Prevalence of non-O157 STEC in pre- and post-grazing fecal samples from sheep (CA) and goats (MN) collected from field trials of integrated crop-livestock systems (2020–2022).

Pathogen in fecal samples	Sheep (CA)			Goats (MN)	
	Pre-graze	Post-graze	Control	Pre-graze	Post-graze
non-O157 STEC	27.5% (11/40)	26.5% (18/68)	35.0% (14/40)	14.7% (11/75)	23.4% (18/77)

#### Diversity of fecal microbiome compositions

**Fig 1:**
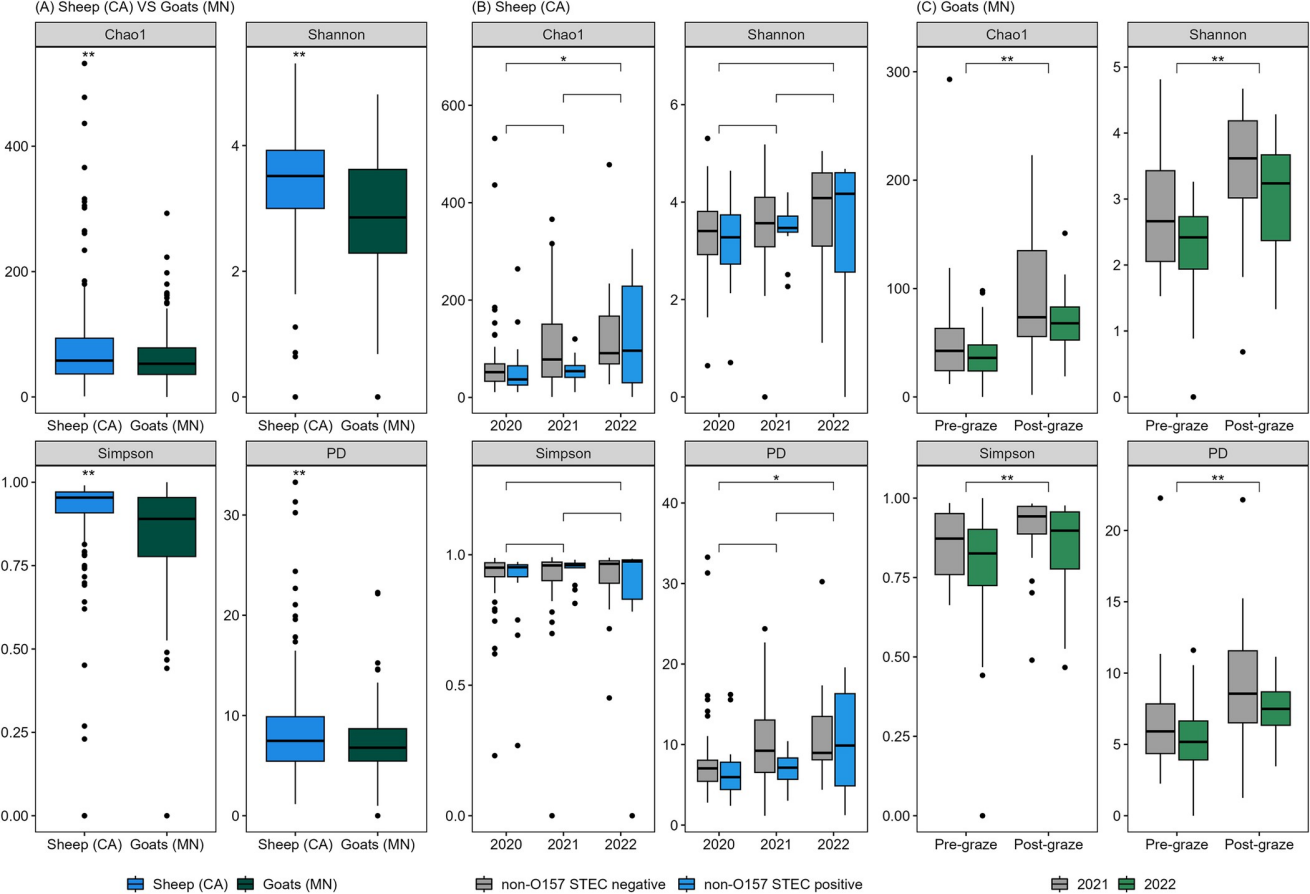
Alpha diversity (Chao1, Shannon, Simpson, and Faith’s phylogenetic diversity (PD)) in fecal samples collected from field trials of integrated crop-livestock systems in California (CA) and Minnesota (MN). Comparisons between (A) sheep (CA) and goat (MN) fecal samples, (B) years (2020–2022) and the presence of non-O157 STEC in sheep feces, and (C) years (2021–2022) and pre- and post-grazing status in goat feces (**p < 0.01, * *p* <0.05).

All four alpha-diversity metrics (Chao1, Shannon, Simpson, and PD) showed significant differences between sheep (CA) and goat (MN) fecal samples (*p* < 0.01). The sheep fecal samples showed higher alpha diversity than the goat fecal samples (Fig 1A). Within sheep fecal samples, the year (2020–2022) and non-O157 STEC presence showed significant associations with Chao1 (*p* = 0.02) and PD (*p* = 0.03) individually. However, when considering both effects simultaneously, only the effect of the year remained significant, with 2022 showing significantly higher Chao1 and PD values than 2020 (*p* = 0.02) (Fig 1B). On the other hand, within goat fecal samples, the year (2021–2022) and pre- and post-grazing status showed significant associations with all four alpha-diversity metrics (*p* < 0.01). When both effects were considered together, the difference in abundance was mostly attributed to pre- and post-grazing status (*p* < 0.001) (Fig 1C).

**Fig 2:**
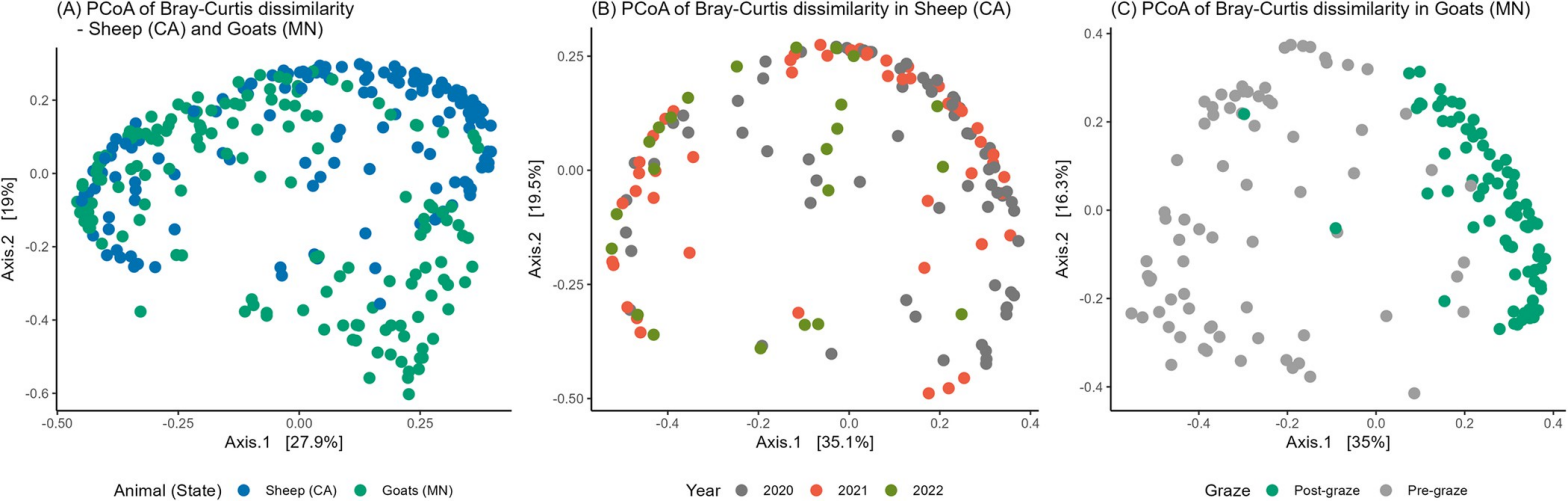
Beta diversity with Bray-Curtis dissimilarity using principal coordinate analysis (PCoA) of fecal samples collected from field trials of integrated crop-livestock systems in California (CA) and Minnesota (MN). Comparisons between (A) sheep (CA) and goat (MN) fecal samples, (B) sheep feces from different years (2020–2022), and (C) pre- and post-grazing status in goat feces.

The beta diversity of sheep and goat fecal samples was significantly different according to both Bray-Curtis dissimilarity (*p* < 0.01) (Fig 2A) and unweighted UniFrac at the ASV level (*p* < 0.01) (S1A Fig). Within sheep fecal samples, both Bray-Curtis dissimilarity (Fig 2B) and unweighted UniFrac significantly differed by year (S1B Fig) (*p* < 0.01). Additionally, unweighted UniFrac was significantly different between samples with and without non-O157 STEC and between the grazing and control groups (*p* < 0.01). Notably, the year effect predominantly explained the variance (R^2^ = 0.05, *p* = 0.001) in the multivariable model when evaluating unweighted UniFrac, with all three variables in the model showing significant differences (*p* < 0.01). Within goat fecal samples, significant differences were observed in both Bray-Curtis and unweighted UniFrac (*p* < 0.05) for the variables of year, pre- and post-grazing status, and the presence of non-O157 STEC. Particularly, the effect of pre- and post-grazing status highly explained the variance in the univariable model (R^2^ = 0.26, *p* < 0.01) with Bray-Curtis dissimilarity (Fig 2C). Interestingly, this effect explained less variance with the unweighted UniFrac (R^2^ = 0.12, *p* < 0.01) (S1C Fig). In the multivariable model with all three variables, the presence of non-O157 STEC became non-significant with the unweighted UniFrac.

#### Differential abundance analysis of fecal samples

**Fig 3:**
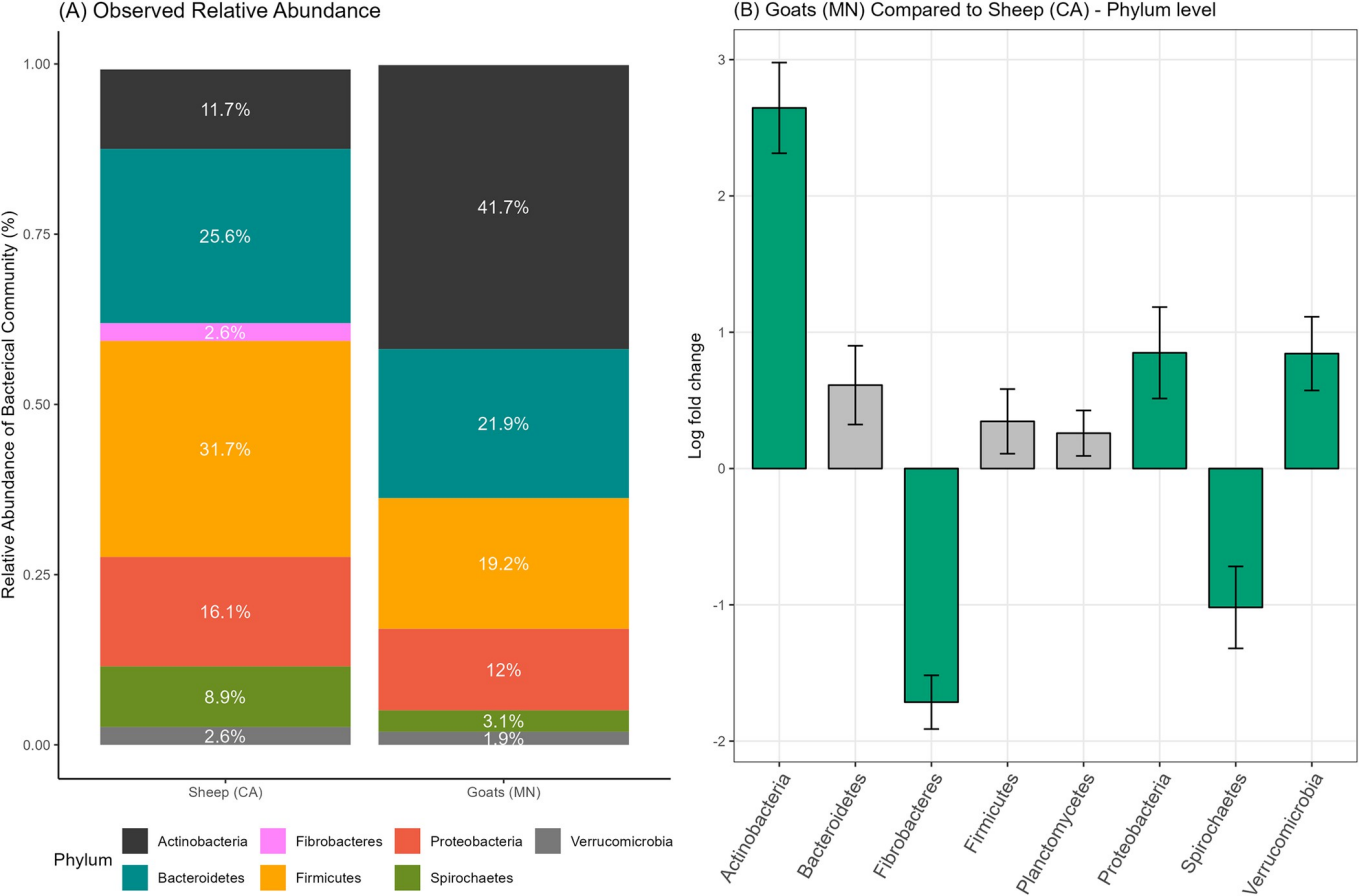
Differential abundance analysis between sheep and goat fecal samples collected from field trials of integrated crop-livestock systems in California (CA) and Minnesota (MN). (A) Observed relative abundance at the phylum level, (B) log fold changes in differential abundance at the phylum level using ANCOM-BC2. (Bar plots highlighted in green indicate significant differences between two groups with a significance level of *p* < 0.05).

Of the 3,022 ASVs observed in fecal samples, 1,013 were shared between sheep and goat fecal samples. Goat fecal samples had a notably higher relative abundance of Actinobacteria (41.7%) than sheep fecal samples (11.7%), which was consistent with the ANCOM-BC2 results showing significantly more Actinobacteria in goat fecal samples (*p* < 0.01). In contrast, Fibrobacteres and Spirochaetes were less commonly observed in goat fecal samples (<0.1% and 3.1%, respectively) than in sheep fecal samples (2.6% and 8.9%, respectively) (ANCOM-BC2, *p* < 0.01) (Fig 3).

**Fig 4:**
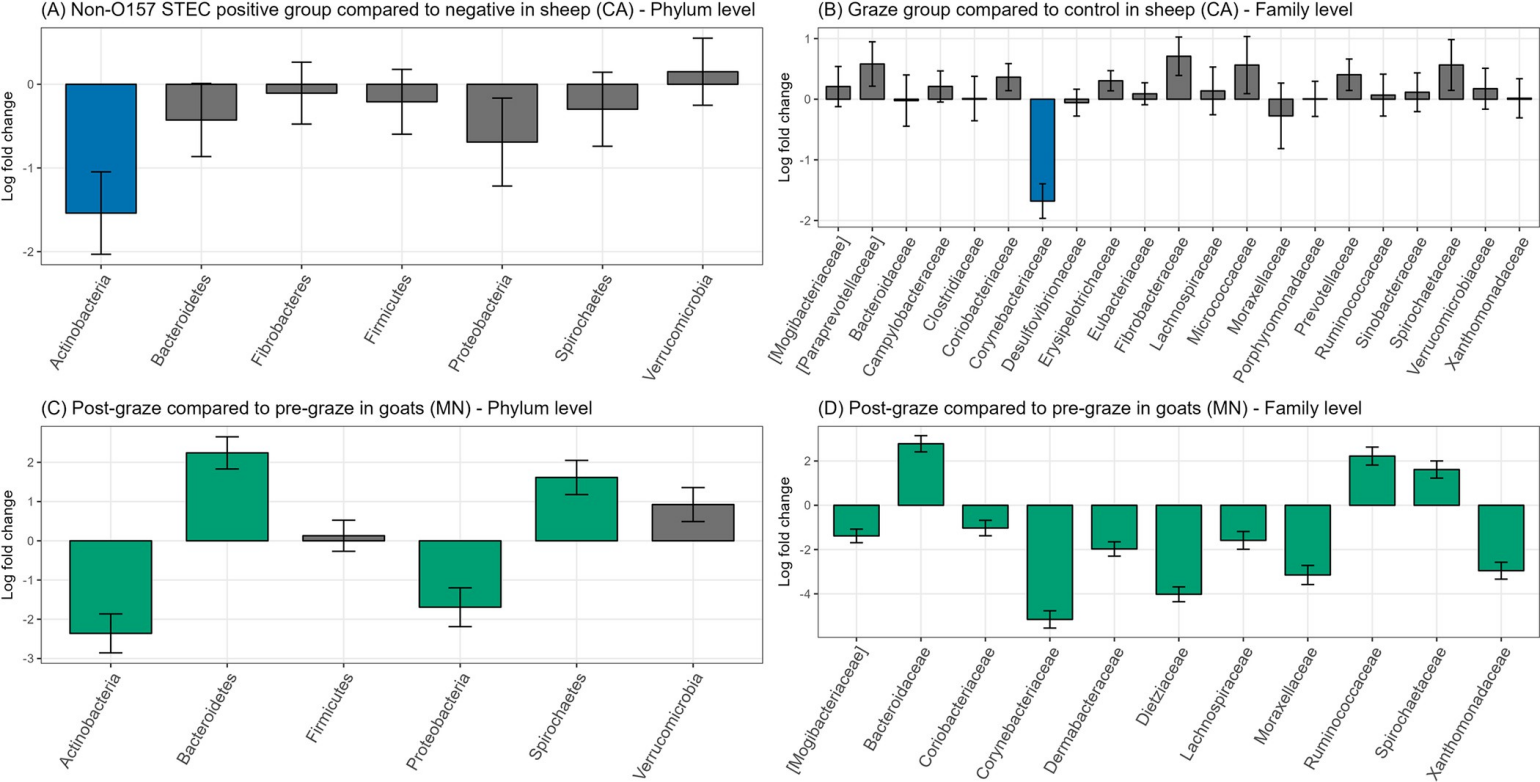
Differential abundance analysis within sheep and goat fecal samples collected from field trials of integrated crop-livestock systems in California (CA) and Minnesota (MN). Log fold changes in differential abundance using ANOCM-BC2 (A) at the phylum level comparing non-O157 STEC positive and negative groups in sheep, (B) at the family level comparing grazing and control groups in sheep, (C) at the phylum level, and (D) at the family level comparing pre- and post-grazing groups in goats. (Blue-colored and green-colored bar plots depict taxa showing significantly different abundance between the compared groups, *p* < 0.05).

When evaluating sheep fecal samples after adjusting for the effect of year, the non-O157 positive fecal samples had a significantly lower abundance of Actinobacteria (log fold changes (LFC) = -1.54, adjusted *p* = 0.01) compared to the non-O157 STEC negative samples (Fig 4A). At the family level, *Corynebacteriaceae* was significantly less frequent in the grazing group than in the control group (LFC = -1.68, adjusted *p* < 0.01) (Fig 4B). Within the grazing group, excluding the samples from the control group, non-O157 STEC-positive fecal samples also showed a significantly lower abundance of Actinobacteria (LFC = -1.68, adjusted *p* < 0.01).

After adjusting for the effect of year, post-grazing goat fecal samples had a significantly lower relative abundance of Actinobacteria (LFC = -2.5) and Proteobacteria (LFC = -1.8) and significantly more Bacteroidetes (LFC = 2.0) and Spirochaetes (LFC = 1.5) than pre-grazing goat fecal samples (adjusted *p* < 0.01) (Fig 4C). The observed relative abundance of Actinobacteria in pre-grazing samples was as high as 60%. However, the abundance in post-grazing samples decreased to 24%, with Bacteroidetes (38%) becoming the predominant phylum. Unlike sheep fecal samples, the abundance of several taxa at the family level (i.e., 12 taxa) significantly differed between the pre- and post-grazing groups (Fig 4D). Among them, the abundance of *Corynebacteriaceae* (LFC = -5.34) decreased the most after grazing, aligning with the findings for the grazing sheep and control sheep (Fig 4B). Unlike sheep fecal samples, no taxa showed significant associations with the presence of non-O157 STEC in goat fecal samples.

### Soil samples

#### Presence of generic *E*. *coli* (gEc) and non-O157 STEC in soil samples

The presence of gEc (%) in each treatment (i.e., one-third of randomly selected samples from each treatment) is summarized in Table 2. Over three years (2020–2022) of trials in both CA and MN, soil samples from the graze CC treatment were more likely to be gEc positive (65.8%, 98/149) than samples from the fallow (29.0%, 38/131) or non-graze CC treatments (30.4%, 41/135). Fifteen soil samples (3.6%, 15/415) tested positive for non-O157 STEC, with the majority (66.7%, 10/15) being from the graze CC treatment. All the non-O157 STEC-positive isolates were found in the spinach/cucumber fields (CA Trial 2 and MN Trial 3).

**Table 2:** Presence (%) of generic *E*. *coli* in soil samples^+^ in each treatment group (fallow, non-graze CC, and graze CC) collected from field trials of integrated crop-livestock systems in California (CA) and Minnesota (MN) (2020–2022).

Trial	Year	Cash Crop	Treatment		
			Fallow	Non-graze CC	Graze CC
CA Trial 1*	2020	Tomato	43.6% (7/16)	52.9% (9/17)	56.5% (13/23)
	2021	Tomato	20.0% (4/20)	12.5% (3/24)	92.0% (23/25)
CA Trial 2*	2021	Spinach	34.8% (8/23)	38.1% (8/21)	63.3% (14/22)
	2022	Cucumber	33.3% (9/27)	25.9% (7/27)	66.7% (18/27)
MN Trial 3	2021	Spinach	19.0% (4/21)	14.3% (3/21)	52.4% (11/21)
	2022	Cucumber	25.0% (6/24)	44.0% (11/25)	61.3% (19/31)
Total			29.0% (38/131)	30.4% (41/135)	65.8% (98/149)

*CA Trial 1—tomato field (2020–2021) and CA Trial 2—spinach/cucumber field (2021–2022) were in the same facility but planted in different plots.

^+^ One-third of collected soil samples were randomly selected from each treatment group.

#### Diversity of soil microbiome compositions

**Fig 5:**
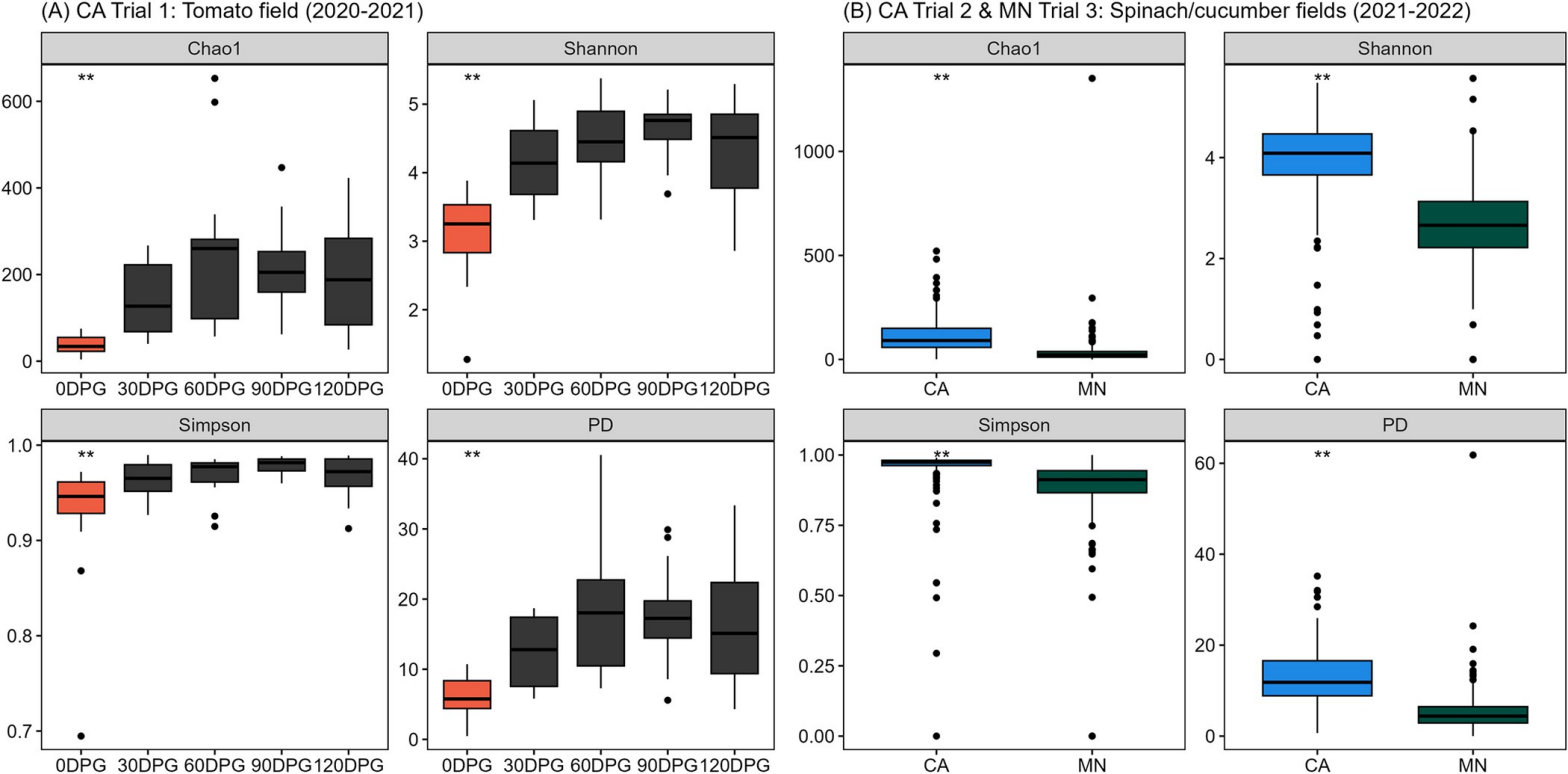
Alpha diversity (Chao1, Shannon, Simpson, and Faith’s phylogenetic diversity (PD)) in soil samples collected from field trials of integrated crop-livestock systems in California (CA) and Minnesota (MN). (A) Sampling day (i.e., day post-grazing (DPG)) effect in the CA Trial 1 tomato field, (B) State effect in spinach/cucumber fields (CA Trial 2 and MN Trial 3) (2021–2022) (**p < 0.01, * *p* <0.05).

The soil samples’ alpha diversity metrics (Chao1, Shannon, Simpson, and PD) differed significantly by sampling days (i.e., DPG) in CA Trial 1. However, the diversity in soil samples from CA Trial 2 and MN Trial 3 varied depending on year, state, and the presence of non-O157 STEC. Specifically, in CA Trial 1, soil samples collected on 0 DPG (i.e., right before the grazing events) showed significantly lower alpha diversities compared to other sampling days (*p* < 0.01), except according to the Simpson index by pairwise comparison (Fig 5A). Alpha diversities from CA Trial 2 and MN Trial 3 were higher in soils from 2021 (compared to 2022) and CA (compared to MN), with no interaction between year and state (Fig 5B). Non-O157 STEC-positive soil samples had lower PD values than negative soil samples (*p* = 0.04). Additionally, none of the alpha-diversity metrics were associated with treatment groups or the presence of gEc for any of the three years (2020–2022).

**Fig 6:**
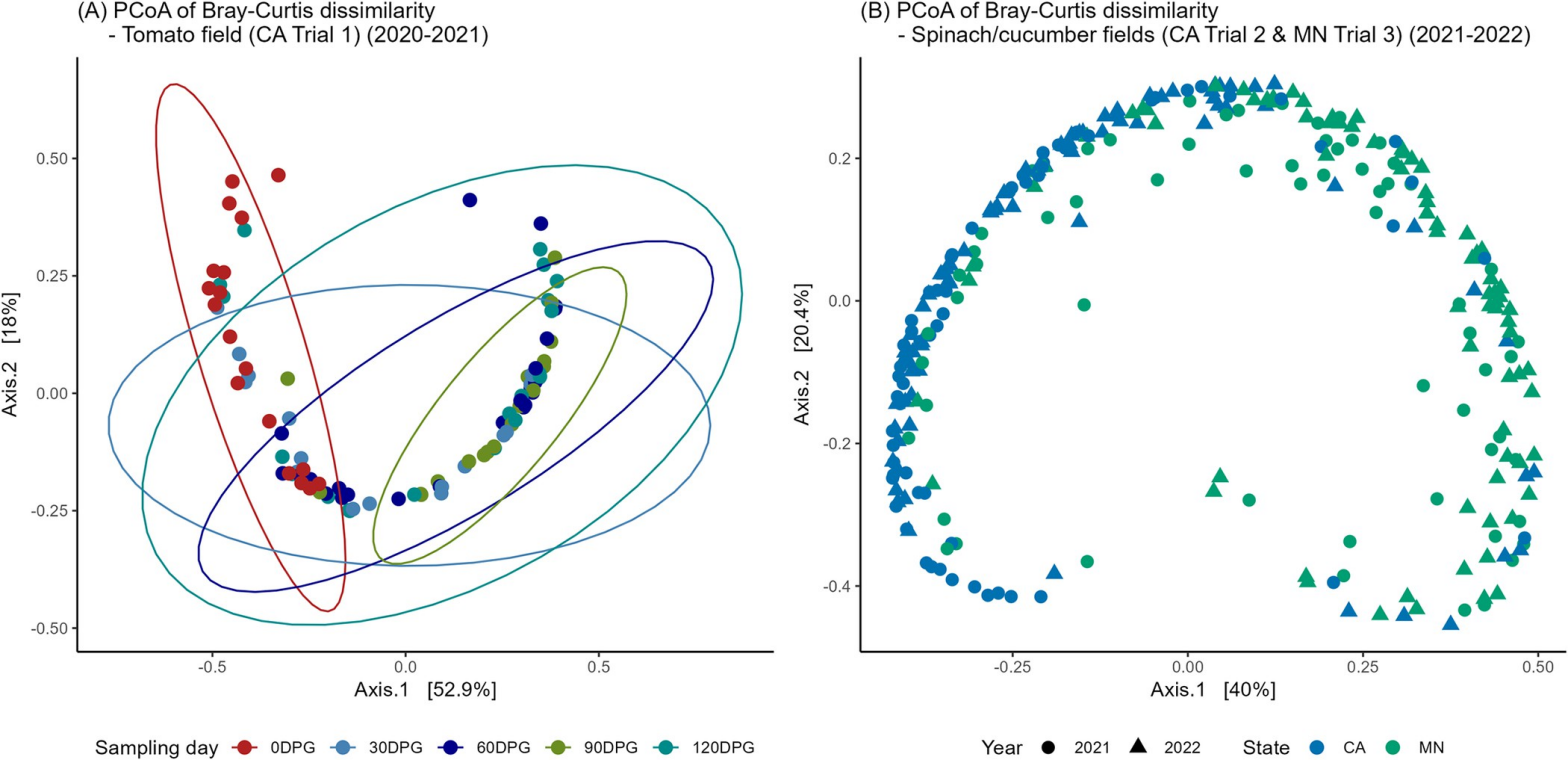
Beta-diversity with Bray-Curtis dissimilarity using principal coordinate analysis (PCoA) of soil samples collected from field trials of integrated crop-livestock systems in California (CA) and Minnesota (MN). (A) Sampling day (i.e., day post-grazing (DPG)) effect in the CA Trial 1 tomato field (2020–2021), (B) state and year effects in spinach/cucumber fields (CA Trial 2 and MN Trial 3, 2021–2022).

In the beta-diversity analysis, significant differences were observed among sampling days (i.e., DPG) in CA Trial 1 for both Bray-Curtis dissimilarity (R^2^ = 0.25, *p* < 0.01) (Fig 6A) and unweighted UniFrac at the ASV-level (R^2^ = 0.14, *p* < 0.01) (S2A Fig). Microbial composition at 0 DPG significantly differed from all other sampling days based on pairwise comparison (adjusted *p* = 0.01) with both metrics. Additionally, unweighted UniFrac was significantly associated with the year (R^2^ = 0.03, *p* < 0.01) effect. On the other hand, in CA Trial 2 and MN Trial 3, both Bray-Curtis dissimilarity and unweighted UniFrac significantly differed by year (*p* < 0.01), state (*p* < 0.01), and the presence of non-O157 STEC (*p* = 0.02) in univariable models. However, in the multivariable model including all three variables, the presence of non-O157 STEC became non-significant, while state explained the most variance in the model (R^2^ = 0.15 with Bray-Curtis and R^2^ = 0.14 with unweighted UniFrac) (Figs 6B and S2B).

#### Differential abundance analysis of soil samples

In CA Trial 1, Actinobacteria at the phylum level showed significantly lower log-fold change on 0 DPG (adjusted *p* < 0.001) than on other sampling days (i.e., 30, 60, 90, and 120 DPG) in pairwise comparisons. At the family level, *Streptomycetaceae* and *Pseudonocardiaceae* were significantly fewer in the soil collected on 0 DPG than on all the other sampling days (adjusted *p* < 0.001). No taxa showed significant differences between treatment groups at the phylum and family level.

**Fig 7:**
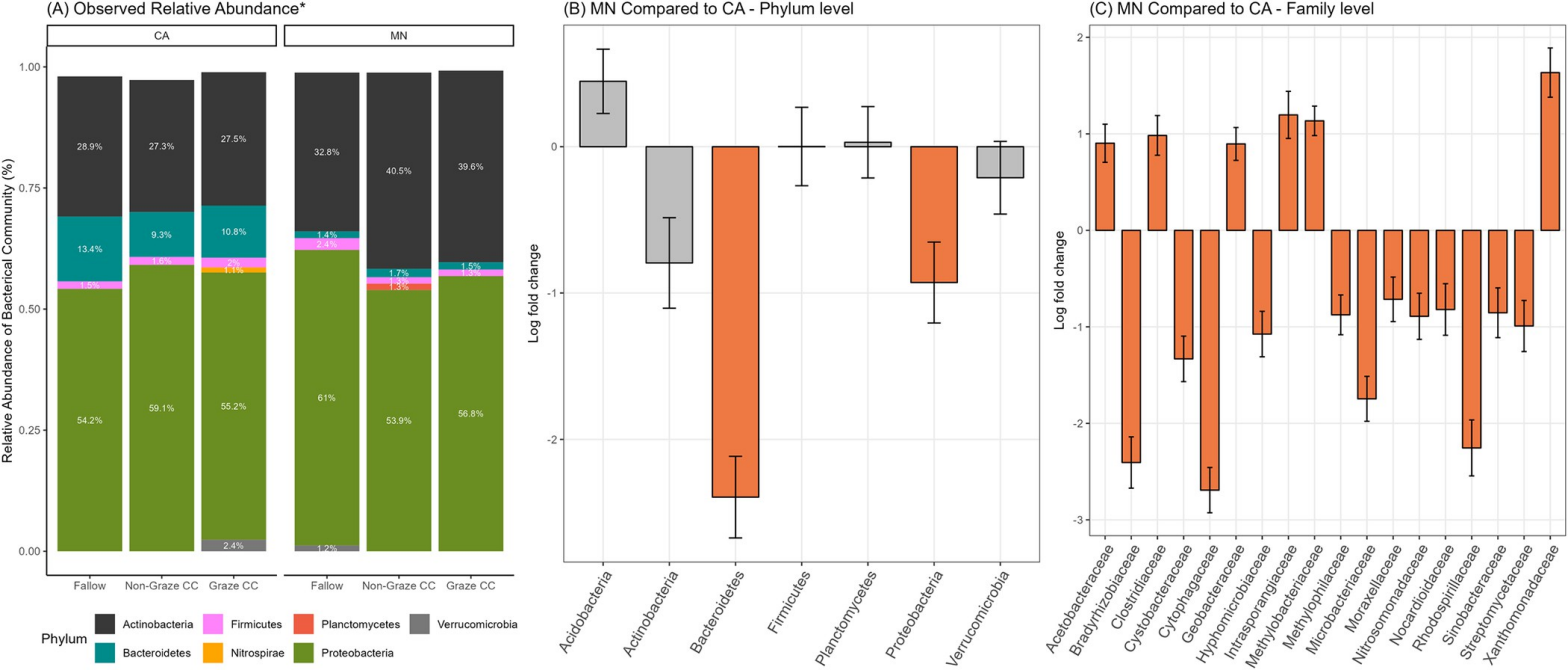
Observed relative abundance and differential abundance analysis of soil samples collected from spinach/cucumber field trials (CA Trial 2 and MN Trial 3) (2021–2022) of integrated crop-livestock systems between California (CA) and Minnesota (MN). (A) The observed relative abundance at the phylum level by treatment group in each state; (B) log fold changes in differential abundance using ANOCM-BC2 at the phylum level; and (C) the family level comparing two states. (Bar plots highlighted in orange indicate significant differences between the two groups, *p* < 0.05). *Relative abundance less than 1% is not depicted in the bar plots.

In CA Trial 2 and MN Trial 3, 16.3% (599/3,683) of ASVs from the soil microbiomes were commonly observed in both states. Between the graze CC treatments in CA and MN, 21.5% (372/1,730) of ASVs were shared, with no differences in observed relative abundance among the three treatments within each state (Fig 7A). Interestingly, the number of ASVs observed in each CA treatment group did not significantly differ (fallow—1,132, non-graze CC—1,167, graze CC—1,428), but in MN, the numbers of ASVs observed in the graze CC and fallow samples were much lower than in the non-graze CC samples (fallow—506, non-graze CC—1,876, graze CC—674). The state had an effect on the differential abundance in the ANCOM-BC2 models, as soil samples from MN exhibited significantly lower levels of Bacteroidetes (LFC = -2.39) and Proteobacteria (LFC = -0.93) at the phylum level than samples from CA (Fig 7B). Fig 7C depicts taxa at the family level that differed significantly between the two states. Treatment had no effect on the differential abundance of the soil microbiome in CA Trial 2 and MN Trial 3.

#### Interactions between post-grazing fecal and graze CC soil samples

The core microbiomes and correlation between post-grazing fecal (n = 145) and graze CC soil samples (n = 141) were evaluated using all data from the field trials in CA and MN (2020–2022). Sheep and goat post-grazing fecal samples consistently contained 9 and 11 ASVs from Bacteroidetes, respectively (Table 3A). Additionally, certain ASVs from Proteobacteria (*Campylobacteraceae* and *Enterobacteriaceae*) were shared among goat post-grazing fecal samples, while the sheep post-grazing fecal samples shared ASVs from Fibrobacteres and Verrucomicrobia. The core microbiome identified in the graze CC soil samples comprised ASVs from Actinobacteria and Proteobacteria. However, the family-level taxonomic classification of shared ASVs in the graze CC soil did not match any observed in the post-grazing fecal samples, except for *Micrococcaceae* (Table 3B). The number of ASVs identified in the core microbiome in graze CC soil collected from CA was 40, whereas in MN, it was 12. None of the taxa at the phylum level were correlated between soil and fecal samples. However, Proteobacteria and Actinobacteria showed positive correlations within fecal samples (ρ = 0.62). In the graze CC soil samples, Bacteroidetes had a strong positive correlation with Actinobacteria (ρ = 0.87) and Proteobacteria (ρ = 0.63), despite the ASVs belonging to Bacteroidetes not being identified as part of the core microbiome in the soil.

**Table 3:** Core microbiomes in (A) post-grazing fecal samples and (B) graze CC soil collected from field trials of integrated crop-livestock systems in California (CA) and Minnesota (MN) (2020–2022).

**(A) Core microbiomes of sheep and goat post-grazing fecal samples.** The taxa in the gray-colored rows represent the core microbiomes commonly observed in both sheep and goats.				
**Phylum**	**Class**	**Order**	**Family**	**Genus**
Actinobacteria	Actinobacteria	Actinomycetales	Micrococcaceae	Unassigned
				Arthrobacter*
Bacteroidetes	Bacteroidia	Bacteroidales	Bacteroidaceae	5-7N15
			Rikenellaceae	Unassigned
			Bacteroidaceae	Unassigned
			Unassigned	Unassigned
			p-2534-18B5	Unassigned**
Firmicutes	Clostridia	Clostridiales	Ruminococcaceae	Ruminococcus
			Lachnospiraceae	Butyrivibrio**
Proteobacteria	Epsilonproteobacteria	Campylobacterales	Campylobacteraceae	Campylobacter*
	Gammaproteobacteria	Enterobacteriales	Enterobacteriaceae	Unassigned*
Fibrobacteres	Fibrobacteria	Fibrobacterales	Fibrobacteraceae	Fibrobacter**
Verrucomicrobia	Verruco-5	WCHB1-41	WCHB1-25	Unassigned**
**(B) Core microbiomes of graze CC soil samples.** The taxon in the gray-colored row represents the core microbiomes of both post-grazing fecal and graze CC soil samples.				
**Phylum**	**Class**	**Order**	**Family**	**Genus**
Actinobacteria	Actinobacteria	Actinomycetales	Micrococcaceae	Unassigned
			Geodermatophilaceae	Unassigned
			Nocardioidaceae	Unassigned
	Thermoleophilia	Solirubrobacterales	Solirubrobacteraceae	Solirubrobacter
			Unassigned	Unassigned
	Rubrobacteria	Rubrobacterales	Rubrobacteraceae	Unassigned
Proteobacteria	Gammaproteobacteria	Chromatiales	Unassigned	Unassigned
			Sinobacteraceae	Steroidobacter
				Unassigned
	Betaproteobacteria	Burkholderiales	Comamonadaceae	Unassigned
			Oxalobacteraceae	Unassigned
	Deltaproteobacteria	Myxococcales	Cystobacteraceae	Cystobacter
				Unassigned
	Alphaproteobacteria	Rhodospirillales	Unassigned	Unassigned
			Rhodospirillaceae	Unassigned
Chloroflexi	Thermomicrobia	JG30-KF-CM45	Unassigned	Unassigned

*Core microbiota only found in goat fecal samples.

** Core microbiota only found in sheep fecal samples.

## Discussion

In this study, grazing had no observable effect on the prevalence of non-O157 STEC in pre- and post-grazing fecal samples in either sheep or goats. Goat fecal samples exhibited significant changes in alpha diversity with differential abundances in specific taxa (e.g., Actinobacteria, Bacteroidetes, and Proteobacteria) between the post-grazing and pre-grazing groups. However, sheep fecal microbial composition did not significantly differ between the grazing and control groups. The presence of non-O157 STEC in sheep fecal samples was associated with a lower abundance of Actinobacteria. A regional difference was observed between soil microbial compositions in CA and MN fields. When the microbial compositions of the post-grazing fecal samples and the graze CC soil were compared, distinct microbial compositions were observed depending on the type of sample, although no interactions were observed between the fecal and soil samples.

Sheep fecal samples had more taxa than goat fecal samples for all four alpha-diversity metrics (Chao1, Shannon, Simpson, and PD). Shabana et al. (2021) also observed higher microbial complexity in sheep compared to goat fecal samples [40]. Such differences may be associated with dietary preference and foraging behaviors, as goats are known to favor browsing while sheep are primarily grazers [41]. Within sheep, the Chao1 and PD metrics were associated with the effects of the year or non-O157 STEC isolation in feces. However, the Shannon or Simpson metrics did not show significant differences due to year or the presence of STEC, suggesting that the diversity difference was primarily driven by changes in species richness rather than distribution or evenness. Within goats, post-grazing fecal samples showed higher alpha diversity than pre-grazing fecal samples, whereas no change was observed within sheep samples. A positive association between a forage-based diet and increased microbial diversity in fecal or ruminal samples has been found in studies in goats, sheep, and cattle [42–44]. Given that grazing periods for goats (an average of 3 days per grazing event, twice a year) were longer than those for sheep (less than 2 days per grazing event, once a year) in this study, the duration of grazing may have contributed to the lack of observed changes in sheep fecal microbial diversity.

A significant difference in fecal microbial composition was observed between sheep and goats in this study. Firmicutes (31.7%) and Actinobacteria (41.7%) were the dominant phyla in sheep and goat samples, respectively. This observation contrasts with other studies in which Firmicutes were the predominant phylum regardless of animal species [16,40,45–47]. In a study comparing fecal microbiota in sheep and goats of the same ages, no significant differences in bacteria abundance were observed [40]. The authors concluded that the similarity was due to both animals being offered the same diet (pellet feed and alfalfa hay) at the same farm. Similarly, cattle and goats from the same farm with similar diets (pasture and hay) had similar microbial compositions in feces [46]. The observed difference in the predominant relative abundance of phylum between sheep and goat fecal samples in this study may be attributed to variations in diet composition and geographical location (the sheep came from a university’s flock in CA, while the goats were from a rental company in MN) before grazing. Indeed, post-grazing fecal microbial composition became similar, as goats showed a slight increase in Firmicutes after grazing events, while sheep from the grazing group exhibited a higher relative abundance of Firmicutes.

In this study, the relative abundance of Actinobacteria in goat fecal samples significantly decreased after grazing on cover crops. A study investigating the fecal bacterial community difference between domestic and feral goats found that the bacterial families overrepresented in domestic goats mostly belonged to Actinobacteria [48]. It concluded that diet might be an important determinant of bacterial community differences, as feral goats browsed 86 species of plants, whereas domestic goats were fed hay and animal feed. Similarly, a higher proportion of dietary concentrates to forage (C:F) contributed to an increased abundance of Actinobacteria, as seen when the differences in ruminal microbiota of goats were investigated under different dietary C:F ratios [49]. A common finding in both sheep and goat fecal samples in this study was that *Corynebacteriaceae*, a family in the Actinobacteria phylum, decreased after the grazing events in goats and was less observed in the grazing group in sheep. However, the function of *Corynebacteriaceae* in small ruminants has not been well documented.

Within sheep fecal samples, the presence of non-O157 STEC was significantly associated with lower Actinobacteria abundance without significant differences in other taxa. Vasco et al. (2021) reported that dairy cattle farms with a higher prevalence of STEC had a lower abundance of Proteobacteria and a greater abundance of Firmicutes [50]. On the other hand, when fecal microbial composition between *E*. *coli* O157-shedding and non-shedding cows were compared on one single farm, the microbial community did not differ by shedding status [17]. Additionally, it has been reported that alpha diversity is associated with the presence of STEC in fecal samples, but the direction and significance of the association varied. Cattle with a higher prevalence of STEC shedding showed a positive association with alpha diversity [50,51], whereas some studies found a negative association or no association [17,52,53]. The present study showed no difference in alpha diversity between non-O157 STEC-positive and negative feces, with the year having a greater effect. However, in the soil microbiome, non-O157 STEC-positive soils from spinach/cucumber fields (CA Trial 2 and MN Trial 3) showed significantly lower PD. Given that only 15 soil samples were non-O157 STEC positive and considering the contradictory results from previous studies, further investigation is needed to determine the microbial composition factors contributing to the presence of STEC. Shedding of non-O157 STEC may vary depending on various factors such as animal species, diet, and intricate interactions among commensal microbial populations in the feces and soil.

The present study showed no significant soil microbial composition changes induced specifically by the graze CC treatment. Similarly, in studies investigating microbial composition changes in soil after manure application, only a few taxa were altered shortly afterward, with no changes in the overall community compositions [22,54]. Similarly, Heuer et al. (2008) found that bacteria introduced with swine manure do not become prominent in the soil [55]. In ICLS farms in Maryland, the relative abundance in the soil bacterial microbiome compositions before and after manure application differed slightly by proportion, with no changes in the ranks of taxa [56]. Rather, soil microbial communities appear to be influenced more by regional differences than by short-term implementation of grazing or manure application. Soils collected from the CA fields showed higher alpha diversity than those from MN fields, and spinach/cucumber fields in MN had lower levels of Bacteroidetes and Proteobacteria than those in CA (CA Trial 2 and MN Trial 3). As such, soil type, including soil pH and texture, is the most important factor in the composition of bacterial communities in soils [57–59].

The core soil microbiome present across the graze CC treatment was dominated by Actinobacteria and Proteobacteria, which is in agreement with other studies [56,60,61]. Only one taxon, *Micrococcaceae*, was common between the graze CC soils and post-grazing fecal samples, with no observed correlations between the fecal and soil microbiomes. Similarly, a study in which feces from concentrated animal feeding operations was applied to fields and the manure-associated taxa in the soil tracked concluded that manure was a minor driver of soil microbiome shifts [60]. Several studies suggest that manure-derived bacteria are not well adapted to survive in the soil and consequently have only a temporary effect on the soil microbial composition [55,62,63].

The present study had some limitations. We implemented relatively short-term grazing events with 1–3-day cycles, determined by the quantity of cover crop biomass in each trial, which varied from year to year given the agricultural conditions (e.g., weather and cover crop maturity). Longer grazing periods may have induced more changes in fecal microbial composition than what we observed. Recent studies have shown that feeding style, age, and geography have significant impacts on the intestinal and rumen microbiota, feeding styles being one of the most influential factors influencing microbiota taxa in goats [64,65]. The period required to see significant changes in gut/rumen microbiota composition by diet change varies from study to study, with an average of 7–20 days in small ruminants [65–67]. Nonetheless, we observed some changes in taxa in goat fecal samples after grazing events, as well as differences in the grazing group compared to the control group in sheep fecal samples. Additionally, we collected fresh fecal samples immediately upon the animals’ arrival in the fields or right after the grazing events due to logistical constraints in restraining the animals in open fields. We have conducted several studies following this protocol [11,26,68]. However, there may be a risk for environmental contamination and changes in the microbial population with exposure to environmental conditions (e.g., temperature, solar radiation, and moisture) [64].

## Conclusion

A few changes in microbial composition due to grazing and the presence of non-O157 STEC were observed in this study. However, no interactions were observed between the post-grazing fecal samples and the graze CC treatment soil, with distinct microbial compositions depending on sample type. While short-term grazing by sheep or goats in ICLS fields has minimal effects on fecal or soil microbiome diversity and composition, the species of grazing animal and regional differences have a significant effect on microbiome dynamics. This indicates that soil contamination by foodborne pathogens and microbiome dynamics is more likely to be influenced by regional and environmental management factors rather than by the agricultural system (i.e., ICLS) itself. To better understand the long-term effects of grazing on diversity and microbial dynamics, longer implementation of grazing under ICLS systems is needed over several years.

## Supporting information

S1 Fig Beta-diversity with unweighted UniFrac using principal coordinate analysis (PcoA) of fecal samples collected from field trials of integrated crop-livestock systems in California (CA) and Minnesota (MN). Comparisons between (A) sheep (CA) and goat (MN) fecal samples, (B) years (2020–2022) with the presence of non-O157 STEC in sheep feces, (C) years (2020–2022) with pre- and post-grazing status in goat feces.
(TIF)

S2 Fig Beta-diversity with unweighted UniFrac using principal coordinate analysis (PcoA) of soil samples collected from field trials of integrated crop-livestock systems in California (CA) and Minnesota (MN). (A) Sampling day (i.e., day post-grazing) effect in tomato field (CA trial 1) (2020–2021), (B) State and year effects in spinach/cucumber fields (CA Trial 2 and MN Trial 3) (2021–2022).
(TIF)
