# Supplementary figures and images for: Exploring the impact of grazing on fecal and soil microbiome dynamics in small ruminants in organic crop-livestock integration systems
Source: PLoS One. 2025 Jan 17;20(1):e0316616. doi: 10.1371/journal.pone.0316616 (PMC11741640; doi:10.1371/journal.pone.0316616)

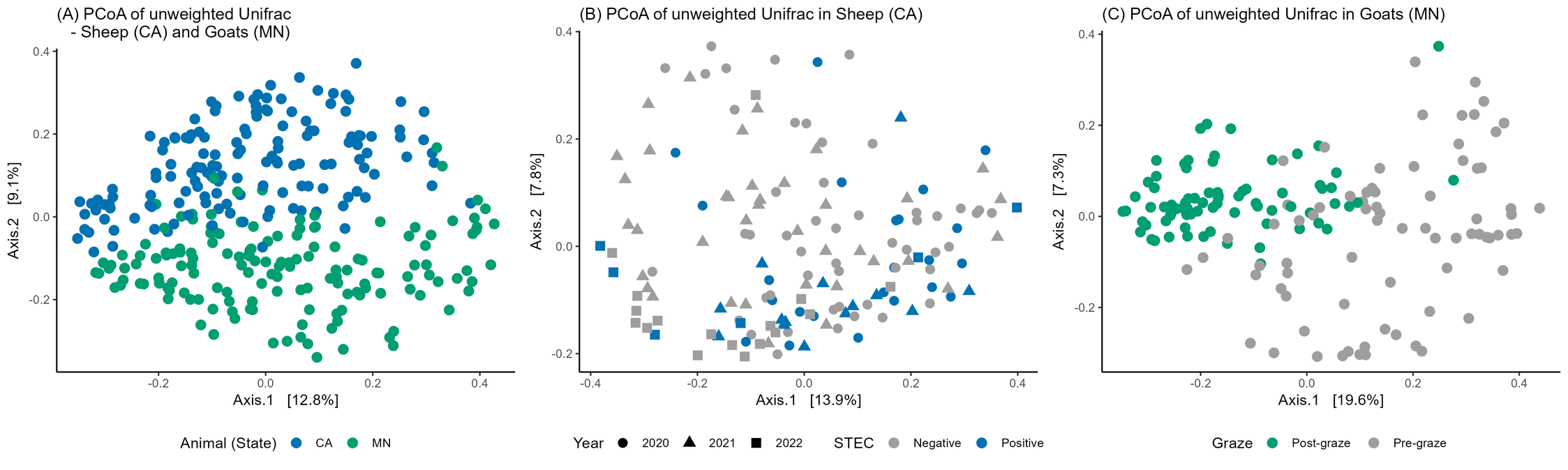

Supplement: S1 Fig — Comparisons between (A) sheep (CA) and goat (MN) fecal samples, (B) years (2020–2022) with the presence of non-O157 STEC in sheep feces, (C) years (2020–2022) with pre- and post-grazing status in goat feces. (TIF) [file pone.0316616.s001.tif]

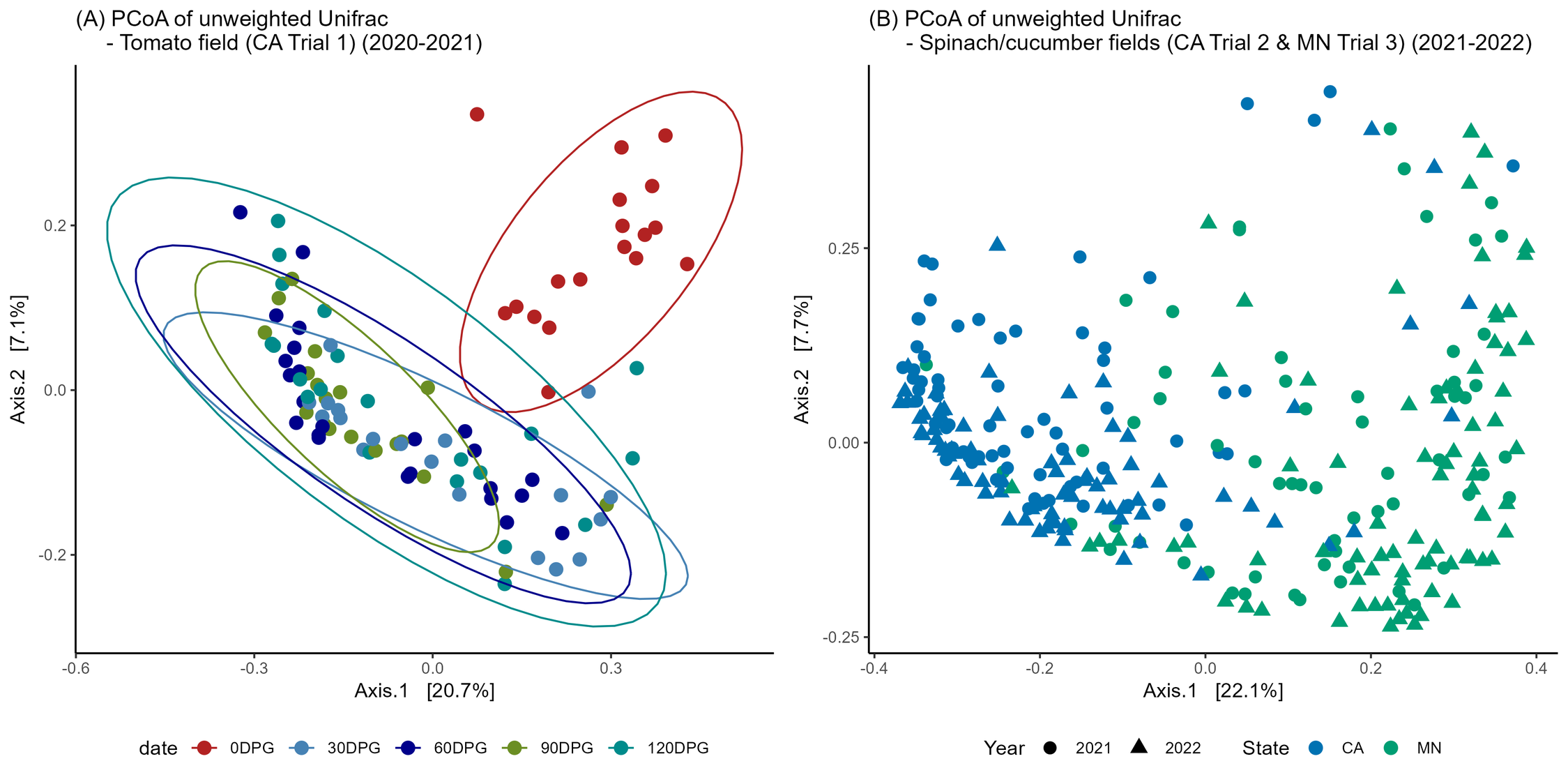

Supplement: S2 Fig — (A) Sampling day (i.e., day post-grazing) effect in tomato field (CA trial 1) (2020–2021), (B) State and year effects in spinach/cucumber fields (CA Trial 2 and MN Trial 3) (2021–2022). (TIF) [file pone.0316616.s002.tif]
